# The preventive treatment of recurrent stone-formation: how can we improve compliance in the treatment of patients with recurrent stone disease?

**DOI:** 10.1007/s00240-015-0842-9

**Published:** 2015-12-14

**Authors:** Dirk Jan Kok

**Affiliations:** Department of Urology, Erasmus MC, Rotterdam, The Netherlands

**Keywords:** Lifestyle, e-health, m-health, Coaching, Stone, Compliance

## Abstract

Whether prevention of Urolithiasis is worthwhile is the outcome of the balance between efficacy of prevention and costs and efforts related of respectively prevention and treatment of a new stone. Well controlled trials demonstrate that effective prevention of new stone formation is possible using medical treatment and lifestyle interventions. In long-term general practice the results obtained with preventive interventions is disappointing. Low and diminishing long-term compliance to the intervention is a major cause for this. Both the long-term aspect and the natural resistance to lifestyle changes contribute to this low compliance. From an analysis of the existing data on trials of preventive interventions and from experiences obtained in other patient groups where lifestyle changes are applied I will make the case that self-empowerment of the patient using m-health lifestyle coaching (a smart phone application) can considerably enhance the level of prevention that is obtained in general practice. In conclusion, I will describe what features will improve usage and efficacy of such an app.

## Introduction

The considerable improvements of methods for stone removal that occurred the last decades may raise the question if efforts for prevention of recurrent stone formation are worthwhile. In addition there are questions of what are successful interventions and how can patients in general practice be persuaded to adhere long-term to such interventions? In this article, I will put forward arguments that support the need for prevention of stone formation, reasons for the differences in efficacy of prevention between controlled trials and general practice, clarify the special role of patient compliance, defend the potential of E-health for supporting patient compliance and describe the essential features of a mobile phone lifestyle coaching application.

Stone formation is a big problem in numbers of patients with a prevalence of around 10 % in the USA [1] and an increasing incidence [2, 3]. It also has a high recurrence rate, 27–50 % within 5 years after a first urinary stone when no specific treatment is given [4, 5]. Given this extent of the problem, researchers have used cost–benefit models to investigate how the costs for prevention, coaching and medication, compared to costs related to stone recurrence, removal and societal costs such as productivity loss. Their conclusion was that the benefits can outweigh the costs [6–11]. It was estimated that for the USA in 1996 medical stone prevention could save $2158 per patient per year [8]. In the UK a single large stone center could save up to 250,000 pound per year at 1998 prices by applying a program of metabolic and nutritional risk factors screening followed by appropriate preventive measures [9]. These numbers relate to secondary prevention, aimed at patients with a history of stone formation.

Of course such studies are based on several assumptions regarding for instance the risk reduction that can be obtained with interventions. For the situation in the USA it has been estimated which criteria should be met in order to make primary stone prevention cost effective [10]. The incidence should exceed 1 %, prevention should achieve a 50 % risk reduction and the yearly cost of prevention should be below US$ 20. Further modelling showed what effect patient compliance has on the results obtained with a simple low-cost preventive treatment, keep fluid intake above 2 L daily [11]. For the total French population cost savings would amount to €49 million assuming 100 % compliance, the dream of every doctor. Savings would amount to €10 million when 25 % of all people is compliant, still a big challenge for a long-term lifestyle intervention. In this model compliance was defined as the percentage of a group that complies long-term with the treatment. Maybe equally important will be how consistent the compliance of the individual patient is. Has the treatment become a part of the normal routine or is it something that is remembered occasionally?

How realistic are the criteria that were used in these models and which factors influence them?

The criterium that the incidence should exceed 1 % is easily met. In the USA the incidence is 7 % for women and 13 % for men [1, 6].

The second criterion of a 50 % risk reduction is also realistic. A relative risk (RR) of 0.5 has been achieved in several randomized controlled trials of stone preventive interventions, as was recently reviewed [12]. RR values range from 0.06 to 0.61 for pharmacologic interventions and from 0.15 to 0.83 for dietary interventions. Although one study reported an increased risk, RR 5.88, in its intervention group receiving the advice to reduce intake of animal protein and increase intake of fiber [13]. Keeping in mind that the number of RCTs of stone preventive treatment is small and the quality of these trials on average is only fair [12], a 50 % reduction of stone recurrence seems feasible. Which intervention will provide the best preventive results is undecided. Something which is clear is that patient compliance is a key determinant for achieving the level of prevention that is promised by controlled trials also in general practice. In an RCT extra funds and time are available for intensive coaching to increase patient compliance. In the RCTs for stone prevention, patients were reminded at least once and up to six times per year to follow their treatment (Table 1). In general practice with its restrictions for time and money the upper limit for coaching may be once a year. There is a need for tools that allow extra coaching in general practice within the limits of time and funding.

**Table 1:** Overview of randomized controlled trials on lifestyle intervention for prevention of stone recurrence

Trial	Intervention	Duration	Patient type	Coaching frequency	RR
Shuster 1992 [54]	Replace soft-drinks	35	37 % 1st, 63 % rec	6 year	0.85
Borghi 1996 [4]	Drinking advice	60	1st with residual fragments	1 + 4 year volume measurement	0.45
Hiatt 1996 [13]	Low protein	42	1st, no residual fragments	2 year	5.88
Kocvara 1999 [14]	Tailored diet	36	1st, 21 % residual fragments	1 year vs. 1, 3 year control group	0.32
Borghi 2002 [17]	Low animal protein, low salt	60	Recurrent, 27 % residual fragment explicit wish for dietary intervention	1 + 4 year volume measurement	0.45
Sarica 2006 [55]	Drinking advice	12	Recurrent	2, 3 year	0.15
Dussol 2007 [56]	High fiber/low animal protein	48	Recurrent	3 year	0.83/1

The criterion that costs of prevention must be below US$20 is a challenge. Of course, the figure was derived using a low estimate for stone incidence of 1 %. The actual incidence is higher what will raise the figure. However, this cost factor appears to exclude pharmacologic treatment. This leaves dietary intervention where the costs are dictated by the dietary changes themselves and by the costs of coaching. It is difficult to assess what it will cost to change from a high risk “Western Style” diet (high intake of animal protein and salt, low intake of fruit and vegetables, low water intake) to one of the advised low risk diets (lower intake of animal protein and salt, higher intake of fruit/vegetables and water, avoiding food with a high oxalate content). Furthermore, this intervention may produce additional societal savings by also lowering the risk for other diseases like those comprised in the metabolic syndrome. In the absence of detailed analysis of this matter I will assume that such changes can be made cost-neutral. This leaves the costs related to the coaching that must accompany lifestyle changes. The approach applied in RCTs, coaching through follow-up visits to the clinic, is very expensive. Again there is a need for low-cost forms of coaching.

Finally the criterion that at least 25 % of the patients must comply with the intervention is also a challenge, especially when it contains elements of lifestyle changes. Kocvara et al. [14] compared the effect of preventive treatment in different medical centers. They confirmed that the patients who showed the lowest compliance to the therapy, as documented by biochemical parameters, had the highest recurrence rate. Comparing the approaches followed in the different centers they concluded that the best compliance was obtained by giving frequent and clear coaching: “the patients find it easier to adhere to a specific dietary regimen than to general instructions.” Thus, a combination of specific instructions and frequent repetition of the dietary counselling seems to provide a good reduction in recurrence.

Data from a 30-year experience of a specialized stone clinic in Chicago show that at best one can retain 70–80 % of patients at each follow up cycle [15]. This required an initial 6-week follow up with 24-h urine collection and subsequent yearly coaching by two physicians based on re-analysis of stone risk factors. Thus even in this dedicated stone clinic setting with a relatively high input of man-power the interest of patients to stay involved with their disease management is rapidly declining. On top of that it is not ensured that the success cases, that is patients who do return, actually adhere stringently to the treatment. In this respect it would help if coaching tools not only stimulate but also register compliance for feedback to both the patient and the doctor.

Overall it appears that low-cost tools for patient coaching and for feedback to the doctor are needed. E- and m-health might provide the needed level of coaching at low cost.

Since a major rationale for using such tools will be to improve patient compliance it is worthwhile to further define the factors that affect patient compliance.

## Patient compliance to stone preventive treatment

Compliance is co-determined by the patient’s motivation. The experience of colic pain can be a good motivator at start. When patients after experiencing a stone episode were asked if they would consider taking medication daily to prevent recurrent pain or a surgical procedure the majority answered yes [16]. In contrast, most of the urologists that were interviewed thought that patients would prefer to avoid medication even in the face of new stone events. The RCT results show that live coaching at a frequency of 1–6 times per year may maintain motivation at a level that delivers the desired risk reduction (Table 1). In general practice some self-coaching tools have been used to support especially the “drinking” advice that is routinely given to patients. One is to ask patients to measure their urine volume every 3 months [4, 17]. The second type of self-coaching is to tell the patient to look at the color of the urine and to drink more when the urine is very dark (Reis-Santos, Lisbon personal communication).

Coaching of dietary advice is more complicated as the dietary advice itself is complex. One mechanism by which dietary advice lowers the risk for stone recurrence is by decreasing the driving force for stone formation, supersaturation. The classic players in this are calcium, oxalate, uric acid, urine pH and volume. A decrease in the urine concentrations of calcium and oxalate and a combination of low urine uric acid concentration plus high enough urine pH lower the risk for calcium oxalate stone formation respectively uric acid stone formation. A first problem is that the urine values are the end result of the cascade of intake, uptake and metabolism-related renal actions. Patients must understand that the intakes of calcium and oxalate effect each-others uptake. Then there is the indirect player, the acid–base balance that amongst others governs urine citrate excretion [18]. A high protein diet provides an acid load which increases the risk of stone formation by decreasing citrate excretion and decreasing inhibition of calcium oxalate crystal agglomeration [19, 20]. Here the information which must be conveyed to the patient becomes even more complicated. The acid–base balance has two dietary sides, dietary acid load and dietary alkali load, plus an individual component, the body size related intrinsic production of acid [21]. Dietary acid load is largely related to protein consumption. Dietary alkali load is largely provided by fruits and vegetables which are sources for organic acids from which bicarbonate can be produced [21]. Thus drinking orange juice, which is acid by itself, provides an alkali source for the human body. Again this is a difficult message to convey. Then there are those food items that on the one hand decrease the risk for stone formation, because they increase urine volume (tea) or are a source of alkali (spinach), while on the other hand, they increase the risk for stone formation by providing extra oxalate. To complicate things further it is advised to consume normal amounts of calcium, a stone component, because it binds oxalate in the gut and thereby lowers crystallization risk in the urine. Many students of medicine cannot reproduce this knowledge correctly in exams taken 1 month after being told this.

This complexity is reflected in the sometimes conflicting data from intervention trials where individual risk factors are singled out. Possibly the least bias is obtained in observational studies of subjects who follow their free choice lifestyle. Several large observational studies are available to show how a low-risk lifestyle could look like [3, 22–24]. The risk for kidney stone formation was found to increase with increasing meat consumption and to decrease with increasing consumption of fresh fruit, fibre from whole-grain cereals and magnesium [24]. In study [24] the hazard ratio was 0.80 in moderate meat eaters (74 and 74 g/day), 0.52 in low meat-eaters (30 and 28 g/day), 0.73 in fish eaters and 0.69 in vegetarians when compared to the cohort with the highest intake of meat (median 135 g/day for men and 127 g/day women). One mechanism to explain these relations may be linked to the acid/base balance of the diet. Table 2 shows the difference in acid load provided by the diets of the different groups in the Turney study, estimated using the approach detailed in [21]. The high meat consumption group is taken as reference for calculating Hazard ratio and acid load difference. Clearly in this study a lower meat intake yields a lower potential renal acid load and a Hazard ratio below 1. Finally, a reduced risk of incident kidney stones during 8 years follow-up was also linked to a greater intake of fiber, fruits and vegetables in a study on 83,922 postmenopausal women [24]. However this study could not confirm this relation for women with a history of stones [25]. An explanation for this may be that the other side of the acid–base balance, the intake of meat and fish, which was not reported in this study, mitigated the alkalinizing effect. The other way around the fact that other epidemiologic studies do no support a relation between a high protein intake and an increased risk for stone formation [23, 26, 27] may be related to mitigation of the acid load provided by protein through an alkali load provided by fruit and vegetables. Finally, complicating all these studies is the fact that there is an intrinsic renal acid load produced by normal metabolism. This production increases with body weight. Increased intrinsic acid production is involved in the relation between obesity and the risk for stone formation [22, 28]. Overall, the focus should be on all aspects of the acid–base balance.

**Table 2:** Net acid load of diets described in [24] plus hazard ratio for stone formation

High meat	Moderate meat	Low meat	Fish	Vegetarian	
1.66	−0.61	−2.25	−4.68	−8.64	Men
−0.87	−2.84	−4.56	−6.72	−9.76	Women
1	0.8	0.52	0.73	0.69	HR

The values for net acid load represent the difference compared to the high meat diet. A negative value denotes less acid (more alkaline) diet

In intervention studies a reduction of the intake of animal protein in combination with a drinking advice has been shown to decrease stone recurrence when compared to patients following a low calcium diet plus the advice to drink more [17]. However, the combination of an advice to reduce animal protein with the advice to maintain an adequate urine volume does not reduce recurrence significantly compared to a drinking advice alone.

Overall the trial data obtained up to now provide some support for the contention that a combination of dietary advice including acid–base balance and intakes of calcium oxalate and salt plus the advice of maintaining a high urine volume may reduce stone recurrence and that the efficacy of the prevention is dictated by patient compliance.

## How can we improve patient compliance to stone preventive therapy?

As outlined above what is needed are tools that provide coaching for taking medication but especially for adapting a desired lifestyle. E- and m-health applications are an option. When constructing such tools it must be kept in mind that the goal is to increase the compliance of the patient to a lifestyle with a low risk for stone formation. Thus the tools must provide correct, detailed, personalized information in a manner that stimulates the patient to actually use the tool.

For the purpose of lowering the risk for stone formation they should provide the user a detailed analysis of the actual diet including water intake with a focus on known risk elements, a comparison versus a diet that poses a low risk for stone formation, advice on improvements, background information on the risk factors for stone formation and a long-time overview. It should also take into account the effects of physical activity and sweat loss, the effect of body size and the effect of the environmental ambient temperature [28]. Finally the analysis should also deal with the complexities that can occur with dietary advice as mentioned before.

Thus, the message that these tools must convey contains seemingly conflicting aspects. Patients are advised to not overconsume foods with a high content of oxalate, including a short list of fruits, vegetables and tea, and at the same time to increase the intake of fruits and vegetables because of their alkalinizing property and to drink enough. What if you want to drink tea? Why is protein an acid load while fruit juice with a low pH poses an alkali load? Furthermore it is told that sweat loss, related to physical activity and ambient temperature, is a risk as it can lower urine volume [29]. On the other hand, data suggest that physical activity reduces the risk for stone formation, possibly because it leads to a lower body weight [30]. The fact that this association between a high physical activity and lower incidence of symptomatic kidney stones could not be confirmed when analyzing three large prospective cohorts [31] may be due to the fact that the risk is mitigated when the sweat loss is compensated by taking extra fluid. How well does the existing supply of E- and m-health tools that aim to support prevention of urolithiasis work in this respect?

As reviewed in this journal recently as of 2014 more than 42 smart phone apps could be found that aim to coach towards lowering the risk for stone formation [32]. Most were designed by patients, a few by health professionals. This development of smart phone apps is still in the early unstructured phase. Most tools provide information to patients or medical professionals, a few provide the option to register the daily intake of water or calcium and some provide suggestions on a low-risk lifestyle. None provide a complete package of lifestyle analysis comprising all risk factors plus up-to date information plus coaching plus feedback to both the patient and the doctor. A few are constructed based on evidence-based information but some provide advice that goes against the current best-practice, like lowering calcium intake. None provide all features that are needed to entice patients to adapt a low risk lifestyle as their lifestyle of free choice. The authors of the review recommend “improving the usefulness of these apps by seeking a ‘quality stamp’ from recognized urological organizations and greater clinician involvement in future app development.” I concur with this statement and would add that in urolithiasis app development there should be two aims: a complete approach as outlined above and a focus on user-friendliness in order to obtain maximal compliance to both using the app and following a low risk lifestyle. For the latter aspect lessons can be learned from behavioral science.

## Coaching by apps, lessons from behavioral science

Many major chronic diseases including urolithiasis and the other diseases grouped under the denominator metabolic syndrome [33] benefit from interventions that target nutrition and physical activity. Although the details of the advice may vary all these interventions in fact try to entice people to follow a balanced lifestyle. All face the same problem of how to approach someone for changing his or her lifestyle. Much can thus be learned from the experiences obtained with patients with renal disease, obesity or even of the general public [34–39]. In addition it must be recognized that a group of urolithiasis patients is a mix of people with a high motivation to start with prevention advice, those who recently experienced a stone event, and of people for whom the memory of the colic pain has faded. The latter must in fact be approached like any other member of the general public with the experience of the stone event being a dormant motivator.

Comparison of interventions aimed at different groups reveals several common “success-features” [39]. One feature is to provide self-management instead of a top-down approach where experts tell subjects what to do [40]. Self-management was found to enhance the results obtained with web-based interventions at the workplace [41, 42]. Examples of self-management for urolithiasis prevention are regularly measuring one’s urine volume and looking at the urine color. Addition of computer aided behavioral change to existing health care consultation can also stimulate self-management and thereby increase the effectivity of the intervention, and can be cost effective but improvement is needed [42–47]. One of the problems that remains to be solved is that usage of the app must not be a barrier by itself. To ensure this the app must adhere to two sets of requirements.

**Fig. 1:**
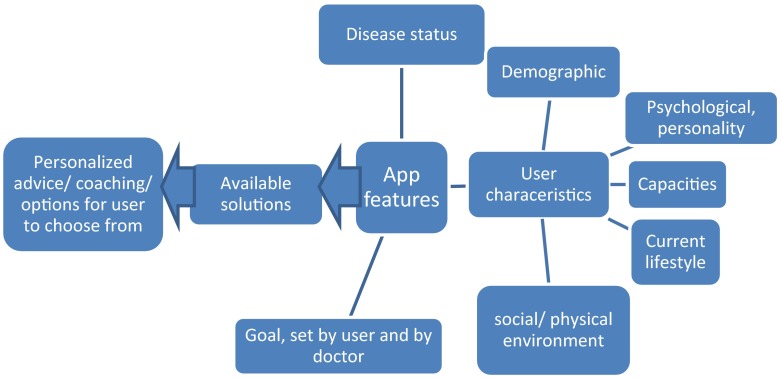
Elements that determine the functional design of a smart phone app

The first set, shown in Fig. 1, concerns the elements which ensure that the app provides the user advice and coaching that is individualized towards their disease status (type of stone, recurrence rate, aberrant urine composition), personality, capacities, current (preferred) lifestyle and social and physical environment. The user characteristics depicted in Fig. 1 relate to intrinsic motivation, internal locus of control (beliefs towards health) and self-efficacy, social norms and support. Personalization of intervention programs towards these user characteristics is key for compliance and change-maintenance. Historically it was thought that lifestyle change requires a mix of motivation, facts, education, action and will power. Once motivated, facts would lead a person to a behavior change that was sustained by periodically re-visiting the facts. It, however, became clear that the induced behavior varies with the individual’s attitudes towards behavior, subjective norms and perceived behavioral control [48]. A parallel may be drawn to experiences with shared decision making. Here patients are asked to participate in the clinical decisions regarding the course of their treatment. A review of the literature on this topic shows that while most efforts have been put in the segment of information provision towards the patient, a neglected barrier is the perception of the patient of really having power to steer the decisions and the standpoint: “the doctor knows best and I am not equipped to make treatment decisions” [49]. Thus while compliance is supported by providing patients free-choice options it is hindered by the inherent uncertainty if the choices are correct. The coaching tool must convince patients that they can make their own correct choices.

Keywords are thus a person’s ‘readiness’ to take action, self-efficacy and perceived barriers [41, 42, 50]. When a population is asked to adapt their lifestyle, only 15 % is ready and willing to start, 40 % is not ready yet and the remainder is even more difficult to convince. Lifestyle adaptation programs that focus on advice content tend to reach mainly the easy 15 %. Customization and intensive coaching are needed to reach also the next 40 % let alone the rest.

Finally, a barrier can also exist on the side of the clinician. Again, a review of the field of shared decision making shows that an important barrier for the implementation of patient decision support tools is indifference on the part of health care professionals who may show less confidence in the content of the advice and have concerns about disruption to established workflows [51]. One suggestion to improve clinician compliance was to provide incentives that reward the use of these interventions. Coaching tools should also benefit the clinician. Benefits could be easy access to long-term lifestyle data and analysis collected by the patient.

Other app features concern the goals set by the doctor and the user. These must on one hand adhere to the most up-to-date evidence based knowledge and support the view of the clinician and on the other hand be flexible enough to meet the individual preferences. For instance advising a patient to eat less meat may be beneficial from a methodological view but will not be efficient when the patient does like to eat meat and consequently has a low compliance to the advice. In this case the better approach might be to provide the option of balancing meat intake with fruit/vegetable intake.

## Requirements for the development of a urolithiasis prevention app

The total set of requirements depicted in Fig. 1 ensures that the user is coached towards an individually determined optimal lifestyle solution.

The experience with patients undergoing renal transplantation may serve as an example. These patients gain weight after transplantation, often becoming overweight or obese. Probable causative factors are a return of appetite, slow return of physical activity, side effects of immune suppressive agents (including appetite for fat) [46]. Furthermore there is individual variation in the risk for weight gain. It is higher in women, in patients with a low economic position, in specific ethnic groups and in people aged >50, [50]. Weight gain in post TX patients can be reduced with changes in diet (negative caloric balance), physical activity and behavior (individual and group-coaching to stimulate, e.g. compliance, coping capacity [36, 52, 53]. A combination of all three plus a high coaching intensity produces the best results, especially when the intervention applies both knowledge transfer (tailored to the individual) and self-teaching [33, 36, 37].

**Fig. 2:**
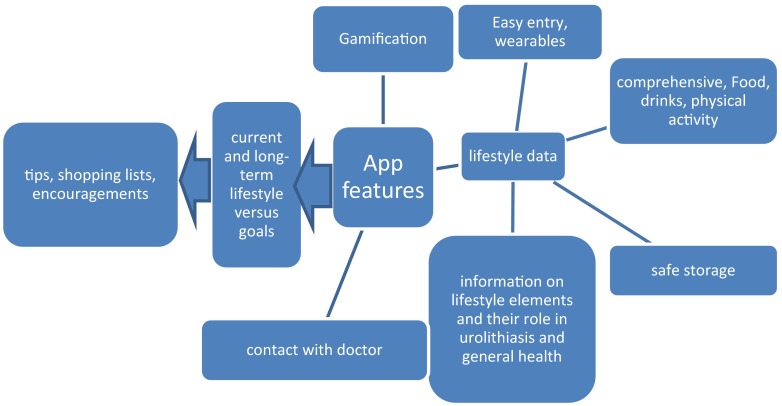
Interface design of a urolithiasis smart phone app

The second set of app features shown in Fig. 2 concerns the app interface and usability. The app must be used at least until the desired lifestyle is no longer felt to be an intervention but has become the natural lifestyle. Data entry must be comprehensive, covering the complete lifestyle but at the same time easy. This is a difficult combination. For registration of physical activity communication of the app with a wearable can be an option. For entry of diet the best balance must be found between completeness of the data (requiring separate entry of all food items that were contained in meals) and ease (entry of complete meals with an estimation on their average composition). Of course the data storage, needed to provide long-term overviews, must be safe. An extra option may be that the app provides a channel for contact with the doctor. Gamification aspects geared to the individual’s social and demographic status may stimulate the use of the app. Finally the advice should be practical, including shopping lists, tips for meals and physical activity and should provide encouragement.

In conclusion, prevention of stone recurrence can be accomplished, coaching patients towards maintaining a high compliance to a low-risk lifestyle is a key factor and new E- and m-health may provide the needed additional coaching.
